# Supplementary figures and images for: Identification and characterization of a novel nematode pan allergen (NPA) from Wuchereria bancrofti and their potential role in human filarial tropical pulmonary eosinophilia (TPE)
Source: PLoS Negl Trop Dis. 2024 Feb 14;18(2):e0011972. doi: 10.1371/journal.pntd.0011972 (PMC10898765; doi:10.1371/journal.pntd.0011972)

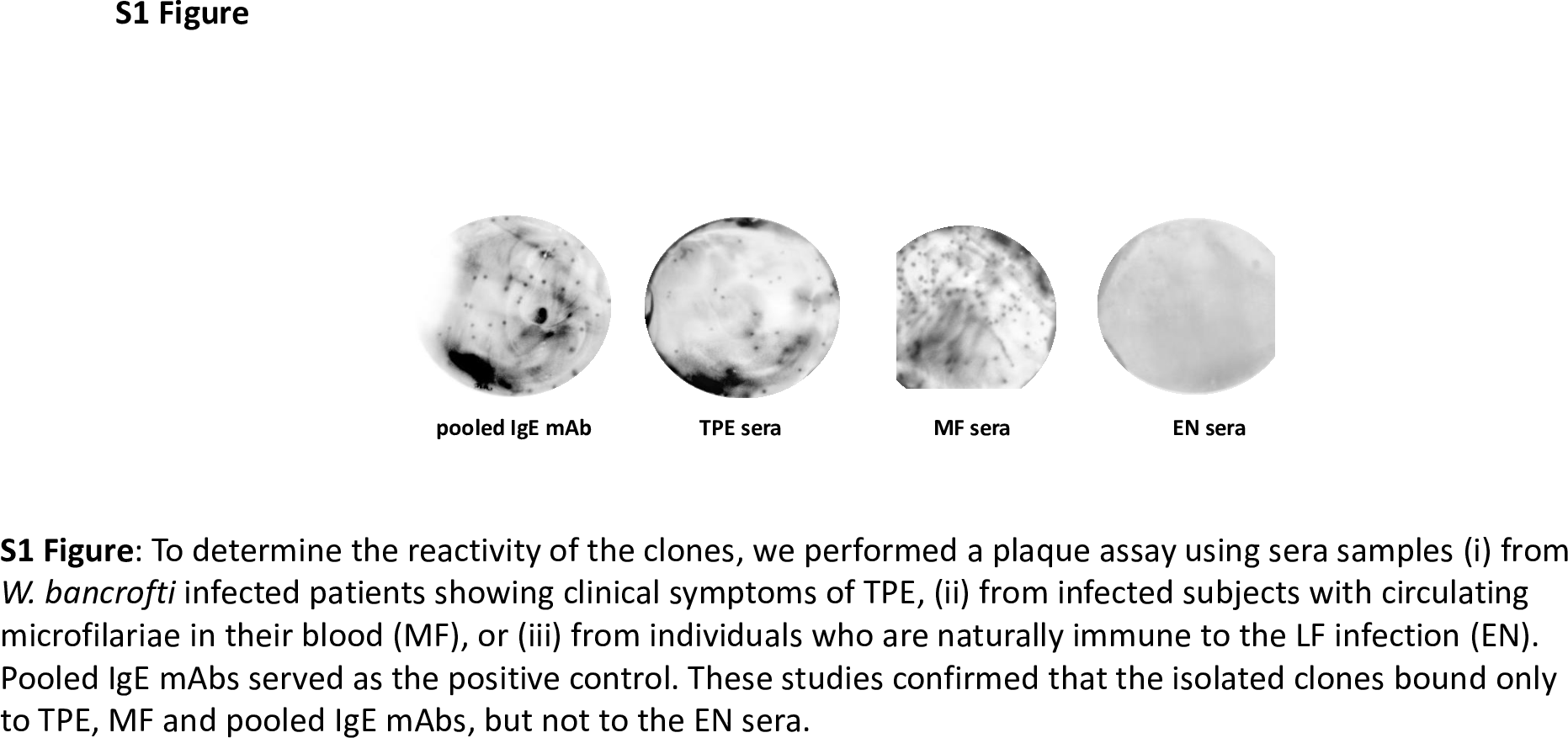

Supplement: S1 Fig — To determine the reactivity of the clones, we performed a plaque assay using sera samples (i) from W. bancrofti-infected patients showing clinical symptoms of TPE, (ii) from infected subjects with circulating microfilariae in their blood (MF), or (iii) from individuals who are naturally immune to the LF infection (EN). Pooled IgE mAbs served as the positive control. These studies confirmed that the isolated clones bound only to TPE, MF and pooled IgE mAbs, but not to the EN sera. (TIF) [file pntd.0011972.s001.tif]
